# Application effect of the online and offline mixed education mode in nursing practice based on the SMCR communication model

**DOI:** 10.3389/fmed.2024.1350975

**Published:** 2024-07-10

**Authors:** Qian Chen, Yanhong Jin, Lixia Zhong, Yingying Li, Li Fu, Weiwei Zhang, Qiuying Xu

**Affiliations:** ^1^Department of Intensive Care Medicine, Beijing Friendship Hospital, Capital Medical University, Beijing, China; ^2^Department of Nursing, Beijing Friendship Hospital, Capital Medical University, Beijing, China

**Keywords:** nursing practice teaching, SMCR communication model, application effect, online, offline

## Abstract

**Objective:**

To explore the application effect of the online and offline mixed teaching mode in nursing practice teaching based on the Source Message Channel Receiver (SMCR) communication model.

**Methods:**

By using the convenience sampling method, 15 nursing students who practiced their internship in the Department of Critical Care Medicine at Beijing Friendship Hospital affiliated to Capital Medical University for 4 weeks, from 1 April 2022 to 31 December 2023, were selected as the experimental group. A total of 16 nursing students who practiced in the same hospital in 2021 were selected as the control group. The control group used traditional teaching mode for teaching, and the experimental group used the online and offline teaching mode for teaching. The theoretical and practical achievements, competency, and teaching satisfaction of the two groups of nursing students were compared and analyzed after the internship.

**Results:**

The theoretical knowledge, operational performance, competence of the nurses, and satisfaction with the teaching in the experimental group were higher than those of the control group (*P* < 0.05).

**Conclusion:**

Based on the SMCR communication model, exploring the online and offline mixed teaching mode plays an important role in the teaching process of nursing practice, which can not only effectively improve the comprehensive performance of nursing students but also help to improve their satisfaction with teaching and has a positive impact on the cultivation of high-quality nursing talents.

## 1 Introduction

With the continuous development of the medical industry in China, the requirements for nursing job are getting higher and higher. Clinical nurses must not only have professional theoretical knowledge but also have strong practical abilities. Therefore, for clinical intern nurses, it is difficult to comprehensively improve their practical ability and comprehensive quality if only the traditional teaching mode is adopted (1). In recent years, with the continuous development of information technology, the traditional teaching mode has been unable to meet the needs of teaching profession. Therefore, the hybrid teaching mode combining offline teaching and online teaching has become more and more popular in major universities. As a brand-new clinical teaching model, it can effectively improve the teaching effect, stimulate students' passion, and help to deepen the interaction between teachers and students. The online and offline mixed teaching mode is based on offline education, incorporates online question and answer (Q&A), uploads nursing teaching videos and materials to the online teaching platform, and carries out multimedia teaching to deepen the theoretical knowledge of intern nurses.

The Source Message Channel Receiver (SMCR) model is an information dissemination model proposed by communication scholar David K. Berlo based on Lasswel's research (2, 3), which is mainly used in the fields of sociology and psychology. The important contribution of the SMCR model is mainly reflected in two aspects: one is to clearly define the four basic components contained in the process of information dissemination and the other is to systematically analyze the key factors that affect the process and effect of information dissemination from different factors (4). The SMCR communication model divides the communication process into four elements: source, information, channel, and receiver, and clearly, and vividly depicts the conditions that affect the information, source, receiver, and channel in order to perform its communication function. Among them, information sources include communication technology, knowledge, attitude, social system, and culture; information includes content, symbols, structure, and composition; channels include various tools for disseminating information, such as sensory organs, light, sound transmission, and modern media such as newspapers, magazines, radio, movies, television, and telephones; receivers are influenced by roughly the same factors as sources, including communication techniques, knowledge, attitudes, social systems, and culture. In the current times, the SMCR model has shifted from being used in the process of information dissemination to explaining the process of educational dissemination. For the purpose of improving the quality of educational dissemination, it is necessary to analyze these four elements. In the process of dissemination in the field of education, the result of educational dissemination information is affected by four factors.

Therefore, to establish a new and efficient nursing practice education method, this study is based on the SMCR model and integrates the online and offline mixed teaching mode to better understand the input characteristics (i.e., source, message, channel, receiver, and destination) of nursing students. We hypothesized that this teaching model would produce better teaching effects and would have high application and promotion value.

## 2 Methods

### 2.1 Participants

By using the convenience sampling method, 15 nursing students who practiced their internship in the Department of Critical Care Medicine at Beijing Friendship Hospital affiliated to Capital Medical University for 4 weeks, from 1 April 2022 to 31 December 2023, were selected as the experimental group. A total of 16 nursing students who practiced in the same hospital in 2021 were selected as the control group. The inclusion criteria adopted were as follows: nursing students who were interns in the Department of Critical Care Medicine and those who provided informed consent and willingness to participate in this study. The exclusion criterion set was: nursing students who take leave for more than 1 week during the internship. The two groups of nursing students completed the nursing practice tasks in the Department of Intensive Care Medicine. There was no statistically significant difference between the two groups in terms of gender, age, and education background (*p* > 0.05), and the practice goals of the two groups were the same, based on the “Nursing Graduation Practice Handbook” for intern nursing students, including the teaching goals of basic nursing skills and the practice goals of the Department of Critical Care Medicine. Through the combination of theory and practice, nursing students can master various clinical critical care skills, cultivate and improve nursing students' clinical thinking ability, post-competency and communication skills, and so on, as well as encourage them to apply knowledge to clinical practice. The two groups are compared using the abovementioned criteria.

### 2.2 Interventions

The control group adopts the traditional teaching mode, and the critical care clinical teachers formulate the nursing student internship plan according to the internship content in the clinical practice outline of the college and adopt the one-to-one teaching mode to complete the internship tasks with the nursing students, that is, the traditional nursing practice mode. During the 4 weeks after the intern nurses entered the department, they received theoretical lectures on the department environment, relevant rules and regulations, precautions, knowledge, and skills of common diseases in the Department of Critical Care Medicine; received lectures on basic nursing operation techniques, tracheal intubation coordination techniques, common critical medical equipment usage techniques, along with other relevant techniques, and checked the interns' mastery on the next day.

On the basis of the control group and the previous theoretical model, the experimental group constructed an online and offline mixed education mode in nursing practice based on the SMCR communication model and implemented this clinical nursing teaching model. The details of the online and offline mixed education mode in nursing practice are as follows:

Formation of the research team: The research team is led by the chief nurse of the department, and the team members include the head nurse of each ward, the head teacher, and the backbone of nursing. The team constructs the teaching content and evaluation indicators according to the requirements of the full-time and specialist practice manuals, mainly including basic nursing skills, operation ability, specialist nursing operation, nursing document writing ability, language expression ability, nursing etiquette, and interpersonal communication ability. The person in charge of the project is the leader of the nursing profession in the Department of Critical Care Medicine. He is responsible for establishing the nursing practice education model, arranging for members to participate in teaching and training regularly, and applying the constructed nursing practice model to the intern nursing students of the Department of Critical Care Medicine. In addition, the teaching inspection team is composed of three to four head nurses and responsible nurses, who inspect the teaching quality from time to time.

**Figure 1:**
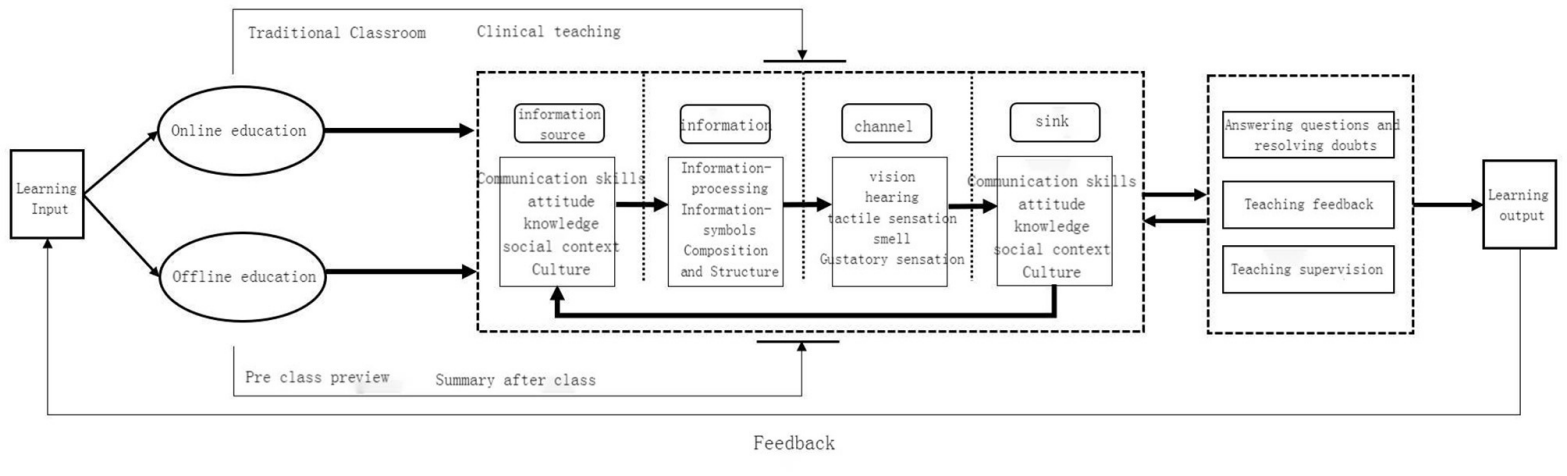
The framework for using the online and offline mixed teaching mode for nursing practice based on the SMCR model.

The online and offline mixed teaching mode in nursing practice based on the SMCR model (see Figure 1 for the framework) was conducted in this study. The specific operation procedures are as follows:

(1) The first step involves clarifying the qualifications of the information source and understanding the characteristics of the information sink. Clinical nursing teachers are the gatekeepers of information and the senders of teaching information. Teachers' attitudes, abilities, knowledge, and social and cultural backgrounds could affect the effectiveness of information transmission. This study requires clinical nursing teachers in the Department of Intensive Care Unit (ICU) to have: have a bachelor's degree or above; have a nursing title or above; have more than 5 years of work experience in ICU; have mastered language expression, organization and management, information collection and processing, interpersonal communication, and other teaching abilities; have proficiency in basic nursing technical operations, specialized technical operations, and specialized theoretical knowledge in intensive care medicine; have proficiency in the clinical application of nursing procedures; have rich teaching experience and a correct and responsible teaching attitude; be able to skillfully use various teaching methods; and be able to master online teaching methods proficiently. When designing the study, the members of the research team first reviewed the general information about the candidate teachers' educational background and work experience. Then, five to six head nurses and primary nurses would be invited to anonymously evaluate these candidate teachers to ensure that the teachers who eventually undertook the teaching tasks met the above nine characteristics and qualifications.
  The receiver is the receiver of clinical knowledge, mainly considering the acceptance ability and attitude of nursing students.
(2) The second step involves benchmarking information elements and refining the content of clinical courses. In the SMCR model, information is the central link of information dissemination, and the composition and structure of information content, symbols, and processing methods all affect the dissemination effect of information. Based on this dissemination effect, the focus of the research is on an online and offline mixed teaching mode, the composition and structure of which required that the content of the nursing practice course in the ICU be practical, complete, and close to the clinic; key and difficult points are highlighted; the content is complete; the goal is clear; the content of the online and offline courses is well-connected; the class schedule is reasonable; in terms of symbols and processing, PPT, paper materials and other pictures, text, video, audio, and other symbols used in the course are clear; quizzes, homework, exams, and other learning resources are abundant; and the scientific nature and difficulty of assessment and evaluation are appropriate. For example, regarding the production of online teaching videos, you should pay attention to the following points:

Appropriate duration: It is best to control the time of online video courses within 5–15 min, and the content should be as short and concise as possible. Rich in content: It is best to use some novel, suspenseful, and problem-based cases or means to attract students, and let students arouse interest in boring knowledge from time to time and learn while watching. Attract attention: Even in the same video course, the scene and tone must be changed frequently, including the tone of the teacher and teaching aids, so that students can further pay attention to knowledge according to different environmental factors. Improving motivation: It is necessary that the design of online video course content enhance students' learning confidence and maintain students' desire for success. For example, some challenging situations or practice questions can be set up, which could be announced by the teacher at the end of the video or after class to make them feel “extraordinary” in their abilities. Appropriate difficulty: The difficulty of the course should be appropriate, and it should be easy to understand so as to enhance the confidence of nursing students, allowing them to appreciate the value and joy of learning through short online videos. It is necessary to ensure that each test question is consistent with the content in order to completely engage the nursing students' vision, hearing, touch, and taste.

(3) The third step is to logically combine the channels. Students' five senses play an important role in knowledge exchange, such as visual perception for non-verbal communication, hearing for receiving and explaining information, touch for acquiring knowledge through behavioral contact, and so on. The main information in clinical nursing practice teaching is vision, hearing, touch, and smell, and the research is based on the characteristics of various course information to clarify reasonable and effective channels.
(4) The fourth step is the clinical practice development process. As the primary supervisor, the clinical nursing teacher is responsible for guiding the internship process one-on-one. Before the internship in the ICU, the clinical nursing teacher assesses the situation of the internship nursing students in the department, including their self-study attitude, self-study ability, communication skills, learning interest, online learning conditions, and so on. According to the characteristics of students, the clinical nursing teacher adjusts the content and methods of practice based on the nursing practice outline plan, provides online learning materials for nursing students in advance, and encourages nursing students to prepare course previews well in advance. Teachers conduct online live teaching in accordance with the weekly internship plan. Offline teaching takes place in conference rooms and ICU wards and adopts methods such as case teaching, group discussion, and bedside clinical nursing teaching.
(5) The fifth step is providing feedback. After completing the teaching tasks of the day, the clinical nursing teachers fully understand the learning situation of the nursing students, answer their questions, listen to their suggestions, provide teaching feedback, and correct any shortcomings in the teaching method.

The clinical nursing teacher project team conducts both online live teaching and offline on-site learning activities. Some specific course content and elements are shown in Table 1.

**Table 1:** Contents and elements of online and offline mixed modes of nursing practice education in critical care medicine based on the SMCR communication model.

**Teaching week**	**Course information**		**Online teaching time**	**Teaching form**	**Channel**	**Whether the teacher has the communication technology**	**Whether the course symbol is appropriate and reasonable**	**Whether the students are interested**	**Whether the students are qualified to study**
	**Curriculum theory knowledge**	**Curriculum practice technology**							
First week	Department-related system and internship requirements	On-site introduction and familiarity with the environment	1 h	Live broadcast teaching and offline ward rounds	Visual and auditory	√	√	√	√
	Basic knowledge of common medical instrument use in the ICU	Field learning of common medical instruments (infusion pump, injection pump, nutrition pump, and glucose meter)	1 h	Live broadcast teaching and offline operation	Visual and auditory	√	√	√	√
	Routine care for common diseases in the ICU	Common ICU physical examination techniques	1 h	Live broadcast teaching and offline ward rounds	Vision, hearing, touch, and smell	√	√	√	√
	Fixation and nursing points of common pipelines in the ICU	Practical operation of pipe fixation	1 h	Live broadcast teaching and offline operation	Vision, hearing, and touch	√	√	√	√
Second week	Identification and nursing of abnormal heart rhythms	ECG monitoring technology	1 h	Live broadcast teaching and offline operation	Vision, hearing, and touch	√	√	√	√
	Care of patients with cardiac arrest	Cardiac electrical defibrillation technique	1 h	Live broadcast teaching and offline operation	Vision, hearing, and touch	√	√	√	√
	Knowledge of common ICU	On-site study of rescue vehicle structure and drug list	1 h	Live broadcast teaching and offline operation	Visual, auditory	√	√	√	√
	Classification and establishment of the artificial airway	Tracheal intubation was performed in combination	1 h	Live broadcast teaching and offline operation	Vision, hearing, and touch	√	√	√	√
Third week	Basic knowledge of the ventilator	Installation of the ventilator line	1 h	Live broadcast teaching and offline operation	Vision, hearing, and touch	√	√	√	√
	Airway care for mechanically ventilated patients	The sputum suction technique via a breath tube for intubation	1 h	Live broadcast teaching and offline operation	Vision, hearing, and touch	√	√	√	√
	Airway temperature and dampness	Oral care techniques for critically ill patients	1 h	Live broadcast teaching and offline operation	Vision, hearing, and touch	√	√	√	√
	Chest physiotherapy	Practice of the chest physiotherapy method (vibration sputum detector)	1 h	Live broadcast teaching and offline operation	Vision, hearing, and touch	√	√	√	√
Fourth week	Management of the common alarms on the ventilator	Handling of the common alarms of the ventilator	1 h	Live broadcast teaching and offline ward rounds	Vision, hearing, and touch	√	√	√	√
	Knowledge of the prevention and control of common respiratory-transmitted diseases	Closed-type sputum suction technology	1 h	Live broadcast teaching and offline operation	Vision, hearing, and touch	√	√	√	√
	ICU hospital infection prevention, control, and management	Wear and take off hand hygiene and isolation clothes	1 h	Live broadcast teaching and offline operation	Vision, hearing, and touch	√	√	√	√
	Preventive measures for the “three tubes” infection	Venous blood collection and intravenous infusion technique	1 h	Live broadcast teaching and offline operation	Vision, hearing, and touch	√	√	√	√

### 2.3 Effect evaluation

Nursing interns in the experimental and control groups completed a theoretical assessment, a skill assessment, a nurse competency inspection, and an intern satisfaction survey. The theory assessment is a closed-book assessment method with a full score of 100 points, and the score indicates the pros and cons of their mastery of theoretical knowledge; the skill assessment is overlooked by the teacher responsible for the assessment, and nursing students randomly select basic operations and specialized operations, with 50 points for each operation. The total score is 100 points. The standard nursing operation assessment used in hospitals is adopted as the scoring standard. The higher the score, the better the students' clinical nursing practice performance; the nurse competency assessment uses a nurse competency scale. The ICU nurse competency survey scale compiled by Qiao Anhua has four dimensions and 58 items, including professional knowledge (14 items), professional technology (19 items), professional ability (20 items), and psychological traits (five items). The scale adopts the Likert 5-level scoring method, with a total score ranging from 0 to 232 points—the higher the score, the greater the level of victorious ability. A score of < 116 is considered as a failure, 116–173 is a pass, and 174 indicates good performance. The scale has a content validity of 0.87, and the Cronbach's alpha coefficient is 0.93. Finally, the questionnaire survey was used to evaluate the satisfaction of nursing interns with the practice of teaching and learning. The evaluation content included the rationality of the practice mode, the quality of teaching in the practice mode, the quality of management of the practice mode, and a comprehensive evaluation of the practice mode. Scores ranged from 0 to 10—the higher the score, the higher the overall rating.

### 2.4 Statistical analysis

Statistical Package for the Social Sciences (SPSS) software, version 24.0, was used for data entry and statistical analysis. Measurement data are presented as x ± s, and statistical inferences are made using the *t*-test. The test level was set at α = 0.05, and a *p*-value of < 0.05 was considered statistically significant.

## 3 Results

### 3.1 Comparison of practice theory and technical operation scores between the experimental and control groups

The results of the study revealed that nursing interns in the experimental group had higher theoretical and clinical nursing scores (93.07 ± 4.46 and 95.53 ± 1.85, respectively) compared to the control group (88.81 ± 5.58 and 91.56 ± 2.97, respectively). The group difference was statistically significant at a *p*-value of < 0.05. The specific results are shown in Table 2.

**Table 2:** Comparison of internship theoretical achievements and technical achievements between the two groups.

**Division of groups**	**Theoretical results**	**Operating results**
Experimental group (*n* = 15)	93.07 ± 4.46	95.53 ± 1.85
Control group (*n* = 16)	88.81 ± 5.58	91.56 ± 2.97
*t*-value	2.335	4.439
*p*-value	0.027^*^	< 0.001^*^

^*^P < 0.05.

### 3.2 Competence comparison of ICU nursing students between the experimental and control groups

The results of the nurse's competency inspection highlighted that nursing interns in the experimental group had higher total, professional knowledge, and professional technical scores (133.27 ± 9.95, 32.20 ± 3.00, and 44.60 ± 4.69, respectively) compared to the control group (122.88 ± 7.293, 28.38 ± 3.24, and 41.50 ± 2.76, respectively), with a statistically significant difference between the two groups. The professional skills and psychological characteristics of the nursing interns in the experimental group were similar to those in the control group (*P* > 0.05). The specific results are explained in Table 3.

**Table 3:** Comparison of ICU nursing students between the two groups.

**Division of groups**	**Experimental group (*n* = 15)**	**Control group (*n* = 16)**	***t***	***p***
Total points	133.27 ± 9.95	122.88 ± 7.293	3.331	0.002^*^
Professional knowledge	32.20 ± 3.00	28.38 ± 3.24	3.400	0.002^*^
Professional technology	44.60 ± 4.69	41.50 ± 2.76	2.226	0.031^*^
Specialized skill	43.13 ± 6.19	40.31 ± 5.26	1.370	1.818
Mental trait	13.33 ± 1.54	12.69 ± 1.30	1.262	0.217

^*^P < 0.05.

### 3.3 Comparison of interns' satisfaction with the teaching mode between the experimental and control groups

A questionnaire survey was conducted to assess the teaching satisfaction of interns, with the evaluation content including the rationality of the internship model, the quality of the teaching of the internship model, the quality of the management of the internship model, and the comprehensive evaluation of the internship model. The results exhibited that there were statistically significant differences between the scores of the experimental and control groups in all aspects (*P* < 0.05), and the scores of the experimental group were higher than those of the control group. The results are shown in Table 4.

**Table 4:** Satisfaction of trainee nursing students with the model in the experimental group.

**Division of groups**	**Rationality**	**Quality of teaching**	**Teaching efficiency**	**Management quality**	**Overall merit**
Experimental group (*n* = 15)	8.20 ± 1.08	7.73 ± 0.46	7.80 ± 0.94	8.13 ± 0.83	8.87 ± 1.13
Control group (*n* = 16)	6.20 ± 1.10	5.92 ± 0.31	6.55 ± 0.81	6.96 ± 0.43	7.12 ± 1.12
*t*-value	4.521	5.376	4.712	3.859	4.512
*p*-value	< 0.001^*^	< 0.001^*^	< 0.001^*^	< 0.001^*^	< 0.001^*^

^*^P < 0.05.

## 4 Discussion

Nursing is a highly practical subject, and clinical practice teaching is an important component of cultivating nursing talents. Nursing content is characterized by abstraction, its complex anatomical structure, and many types of therapeutic drugs, making it difficult for nursing students to accurately understand and remember during the internship stage and apply the knowledge they have learned to clinical practice. Therefore, the selection of appropriate teaching methods for nursing practice has a great impact on improving the efficacy of nursing students' practice, cultivating clinical thinking ability, and improving teaching quality (5).

Due to the lack of emphasis on clinical teaching jobs in some hospital nursing departments, there is a lack of scientific and reasonable assessment of the teaching qualifications of teaching teachers. The uneven quality of teaching among some teachers has a direct impact on the clinical practice experience of nursing interns. Some teaching teachers do not have a deep and clear understanding of the teaching profession; they treat the nursing interns as if they were their own helpers, lack the sense of responsibility for the nursing interns, and do not provide clear guidance. Due to the lack of comprehensive and scientific evaluation after students enter the course, some teachers do not fully understand the students' theoretical knowledge, practical operation skills, and personality characteristics; as a result, they adopt a mechanized and repetitive teaching mode, which cannot obtain satisfactory teaching results. Due to the lack of teaching objectives and plans in clinical teaching jobs, some nursing interns are unable to clearly understand the content and skills that must be mastered during the internship stage after entering the department and instead passively follow the teacher's to learn, which limits their efforts to take subjective initiatives to completely engage in learning. Considering that medical students lack clinical practice experience and have a low level of cognition of the clinical nursing process and content, teachers with specific clinical experience should be entrusted to guide and assist nursing interns in completing the established clinical nursing tasks. In this process, the professional quality and teaching effectiveness of the teaching teachers have a direct effect on the improvement of nursing interns' clinical nursing level (6, 7).

In addition, nursing students entering clinical practice can master the disease characteristics and nursing methods of different specialties by rotating through different departments; however, in the actual teaching process, some teachers focus more on basic operations, making it impossible to fully understand and master the various characteristics of different specialties. In light of various problems in the clinical teaching process of nursing interns, as well as the need to cultivate nursing personnel who meet the clinical requirements, it is necessary to further optimize the clinical teaching profession.

The study found that, due to the lack of clinical nursing thinking and the ability of interns, coupled with insufficient nursing technology and communication skills, the interns could not quickly enter the nursing field. Therefore, with a view to improve the working ability of nursing students, it is necessary to collaborate with scientific teaching mode in clinical nursing to improving comprehensive ability and lay the foundation for their future participation in clinical nursing jobs. The traditional teaching mode focuses solely on teaching professional skills and provides less focus on training on intern's comprehensive skills and psychological quality, but the practice has shown that it is necessary to strengthen the teaching methods in all aspects, increase focus on training, pay attention to details, establish dynamic monitoring, and maximize teaching interaction as a way to improve the teaching effect of intern nursing students. In clinical nursing, trainee nursing students not only need to have solid theoretical knowledge but also strong practical skills.

As a brand-new teaching mode, the online and offline mixed teaching mode primarily uses the Internet to carry out education, which effectively promotes the development of nursing education. It offers several advantages, such as greater flexibility, unrestricted time, and location independence. The online and offline mixed teaching mode is an interactive one that allows students to gather the necessary information through the Internet to effectively solve the problems raised by themselves and teachers. This approach enables students to better grasp and understand the knowledge points and use them to enhance students' ability to discover, analyze, and deal with problems (8, 9) while also improving team awareness and self-learning awareness (10), thereby improving teaching effectiveness (11). In recent years, many colleges and universities have been competing to adopt online and offline mixed teaching (12, 13), which has become the current trend (14).

Every teaching system can be regarded as an information dissemination system, and the online and offline mixed teaching mode is no exception. The SMCR model is used not only in the fields of psychology and sociology but also in educational dissemination as a means to explore and optimize. Focusing on the four dimensions of the SMCR model, this study optimizes it by integrating the online and offline mixed teaching mode and discusses its application effect. First, the teaching team on the source side serves as the source of information. In the process of nursing practice courses, there are many factors that influence the source of information. The source's attitude, knowledge, and experience, communication skills, and culture have a direct impact, indicating that the quality and impact of information dissemination bear the main responsibility (15). This study is led by the chief nurse, and the team members include the head nurses of each ward, the head teacher, and the backbone of nursing. A teaching team comprising outstanding members of each ward has been formed, a nursing practice education model has been established, and members have been scheduled to participate in teaching and training regularly to fully ensure the quality of information sources. Second, the message is the central part to information dissemination in the SMCR model, and the composition and structure of information content, symbols, and processing methods all have an effect on the dissemination effect of information: In terms of symbols, they can be verbal or non-verbal; auditory or visual; and meaningful or meaningless (3). The images, text, video, audio, and other symbols used in this research course, such as PPT and paper materials, are clear; learning resources, such as tests, homework, and examinations, are abundant; and the assessment and evaluation are scientific and difficult. In terms of content, for small-scale online courses, the practical content should be organically combined with technology and diverse face-to-face activities such as lectures, experiments, problem solving, project design in order to achieve different teaching objectives, teaching content, and learner characteristics (16). The composition and structure section requires that the content of the intensive care practice nursing practice course be practical, complete, and closely related to the clinic; important and difficult points are highlighted; the content is complete; the goal is clear; the online and offline course content is well-connected; and the class schedule is reasonable. In terms of processing methods, the innovation of new course content for teaching should be considered the cornerstone of the entire nursing practice course. Teaching in nursing practice should be closely connected with student to improve targeted learning outcomes. When considering the influence of channels in the SMCR model, it involves five sensory factors: vision, hearing, touch, smell, and taste. Taste and smell do not play a big role in online teaching but vision and hearing do. However, vision, hearing, touch, smell, and taste all play a role in the offline teaching process.

In this study, the content of online and offline courses was well-connected, and the class schedule was manageable. The course's difficulty was appropriate and understandable, which boosted the confidence of nursing students. Additionally, it is crucial to ensure that the test questions are aligned with the content, ensuring that nursing students feel the value and joy of learning through short online video lessons in order to completely engage their senses of sight, hearing, touch, smell, and taste. Finally, this study found that strengthening teacher-student interaction, student-student interaction, student-media interaction, and teacher-media interaction can effectively promote teaching effects. After completing the online course, combined with the information platform, the teaching teacher can grasp the learning situation of the students at any time, adjust the course arrangement, set evaluation goals according to the platform's exercises, exchanges, homework, tests, etc., and form a learning situation analysis. At the same time, combined with offline practice, discussion, report, and evaluation, the teaching evaluation is finally formed, which lays the foundation for the course design of the next teaching cycle (17).

The results of this study depicted that the test scores, competencies, and teaching satisfaction of nursing students in the experimental group were better than those in the control group (*P* < 0.05). The reason for this is that online and offline hybrid teaching is a teaching method that combines both online and offline instruction, as well as one that spans time and space. Combining the benefits of online and real-world teaching and integrating people into teaching process can result in multi-element and multi-dimensional unified coordination, and students can repeat online learning. Teachers can also answer questions left by students. Students can study independently on the platform, and after completing homework and tests, teachers can answer and correct homework online, which significantly improves teaching quality and efficiency.

## 5 Conclusion

The online and offline mixed teaching mode based on the SMCR communication model can effectively improve the comprehensive performance and the overall level of nursing students in the process of nursing practice teaching, as well as the satisfaction of nursing students with teaching, and cultivate nursing students' learning ability, problem-solving ability, and clinical thinking ability so that their comprehensive quality can be substantially improved and the teaching effect is remarkable. These findings have important guiding significance and reference value for exploring diversified nursing teaching modes and cultivating high-quality nursing talents.

This study also has some limitations. First, the small sample size reduces test efficiency, resulting in no statistically significant differences between the experimental and control groups for some items. Second, due to the difficulty of sample inclusion, the control group used previously collected data and did not abide by the principle of random grouping. It is also worth noting that the SMCR model has inherent limitations. McGuire's theoretical framework of SMCR model guides the pre-production stage of formative research on the target audience, but this theory does not account for subtle changes in the process of practice based on the audience's acceptance of information. This result can lead to discrepancy in the expected teaching progress and the absorption and integration of knowledge by the actual audience. More teaching models combined with SMCR model will be explored in the future to further improve the quality of nursing education.

## Data availability statement

The original contributions presented in the study are included in the article/supplementary material, further inquiries can be directed to the corresponding author.

## Ethics statement

This study was conducted in accordance with the Declaration of Helsinki and approved by the Ethics Committee of the Beijing Friendship Hospital. Written informed consent was obtained from all participants. The studies were conducted in accordance with the local legislation and institutional requirements. The participants provided their written informed consent to participate in this study. Written informed consent was obtained from the individual(s) for the publication of any potentially identifiable images or data included in this article.

## Author contributions

QC: Data curation, Formal analysis, Methodology, Project administration, Visualization, Writing – original draft, Writing – review & editing. YJ: Conceptualization, Data curation, Formal analysis, Methodology, Visualization, Writing – original draft, Writing – review & editing. LZ: Formal analysis, Methodology, Writing – original draft, Writing – review & editing. YL: Formal analysis, Methodology, Project administration, Writing – original draft, Writing – review & editing. LF: Formal analysis, Methodology, Project administration, Writing – original draft, Writing – review & editing. WZ: Formal analysis, Methodology, Project administration, Writing – original draft, Writing – review & editing. QX: Formal analysis, Methodology, Project administration, Writing – original draft, Writing – review & editing.
